# Characterization and Comparison of Intestinal Bacterial Microbiomes of *Euschistus heros* and *Piezodorus guildinii* Collected in Brazil and the United States

**DOI:** 10.3389/fmicb.2021.769965

**Published:** 2021-10-27

**Authors:** Matheus Sartori Moro, Xing Wu, Wei Wei, Lucas William Mendes, Kerry Clint Allen, José Baldin Pinheiro, Steven J. Clough, Maria Imaculada Zucchi

**Affiliations:** ^1^Genetics and Molecular Biology – Biology Institute, State University of Campinas (UNICAMP), Campinas, São Paulo, Brazil; ^2^Genetics and Genomics Conservation Laboratory (UNICAMP), Piracicaba, Brazil; ^3^Department of Crop Science, University of Illinois, Urbana, IL, United States; ^4^Center for Nuclear Energy in Agriculture (CENA), São Paulo, Brazil; ^5^United States Department of Agriculture-Agricultural Research Services, Southern Insect Management Research, Stoneville, MS, United States; ^6^Department of Genetics, Luiz de Queiroz College of Agriculture (ESALQ), Piracicaba, Brazil; ^7^United States Department of Agriculture-Agricultural Research Services, Soybean/Maize Germplasm, Pathology and Genetics Research, Urbana, IL, United States; ^8^São Paulo Agency of Agribusiness Technology (APTA), Piracicaba, Brazil

**Keywords:** distribution, glycine max, insect pests, microflora, stink bugs

## Abstract

**Background:** Herbivorous insects are one of the main biological threats to crops. One such group of insects, stink bugs, do not eat large amounts of tissue when feeding on soybean, but are damaging to the quality of the seed yield as they feed on green developing seeds leading to poorly marketable harvests. In addition to causing physical damage during sucking-feeding activities, the insects can also transmit microbial pathogens, leading to even greater yield loss. Conducting surveys of the insect intestinal microbiome can help identify possible pathogens, as well as detail what healthy stink bug digestive systems have in common.

**Methods:** We used the conserved V4 region of the 16S rRNA gene to characterize the bacterial microbiome of the red-banded stink bug *Piezodorus guildinii* collected in Brazil and the United States, as well as the neotropical brown stink bug *Euschistus heros* collected in Brazil.

**Results:** After quality filtering of the data, 192 samples were kept for analyses: 117 samples from *P. guildinii* covering three sites in Brazil and four sites in the United States, and 75 samples for *E. heros* covering 10 sites in Brazil. The most interesting observations were that the diversity and abundance of some bacterial families were different in the different ecoregions of Brazil and the United States.

**Conclusion:** Some families, such as Acetobacteraceae, Bacillaceae, Moraxellaceae, Enterobacteriaceae, and Rhodocyclaceae, may be related to the better adaptation in some localities in providing nutrients, break down cellulose, detoxify phytochemicals, and degrade organic compounds, which makes it difficult to control these species.

## Introduction

One of the largest threats to crops is insects, either directly through their feeding or indirectly *via* pathogen transmission (Gullan and Cranston, 2014; Hirakuri and Lazzarotto, 2014; EMBRAPA, 2018). An example of important crop-damaging insects is the stink bugs (Hemiptera) and feed by piercing their stylets into the plant host tissue (Esquivel, 2019), which can cause direct injuries, inject toxic substances, or transmit pathogens, reducing host tissue health (Venzon et al., 1999; Gallo et al., 2002; Gullan and Cranston, 2014; Fujihara et al., 2016; Hosokawa et al., 2016). The attack on crops can be very damaging as the insects prefer feeding on the nutrient-rich developing seed. Stink bugs like to feed on legumes such as soybean (*Glycine max* L.), common bean (*Phaseolus vulgaris* L.), peas (*Pisum sativum* L.), and alfalfa (*Medicago sativa* L.), in addition to multiple alternative host plants that might be found within or near an agricultural field (Panizzi et al., 2012).

Throughout their lives, stink bugs can explore several environments and diverse food sources, which can modulate de gut microbiome. As seen in many insects, the bacterial community varies in population size, composition, location, and function within the gut, which can affect the health of the insects, as well as how likely they transmit pathogens to the plant host. Most of the microorganisms that enter into the gut lumen transiently pass through or are eliminated by the host immune system, but some of them remain longer and proliferate in the gut lumen. The wide range of insect associations with intestinal microorganisms is illustrated within Hemiptera (Engel and Moran, 2013; Yun et al., 2014; Lee et al., 2017).

The microbiota of an insect can be strictly hereditary, allowing nutrient supply to the host who lives in a restricted environment (Engel and Moran, 2013). In stink bugs, gut microbiome would be vertically transmitted, these gut symbionts are deposited on eggs every generation and co-evolve with the host and are important for basic host functions such as developmental programs, or essential adaptations. The intestinal microbiome also can be acquired through horizontally transmitted, which are better adapted for host colonization than environmental strains, and can facilitate host adaptation to new niches or environmental conditions. Beyond vertical and horizontal transmission, the environmental microbes can be acquired by feeding, which is advantageous in facilitating host adaptation to new conditions but are not adapted to the host (Kikuchi et al., 2007; Hosokawa et al., 2016; Shapira, 2016).

The absence of endosymbionts in the stink bug gut microbiome slows their growth and increases mortality and sterility since the gut microbiome plays an important role in insect health (Zchori-Fein and Bourtzis, 2011). Furthermore, prolonged interaction with a specific member of the gut microbiota can have an impact on a wide range of host physiologies (Engel and Moran, 2013; Lee et al., 2017).

Several cases of insects resistance by endosymbionts bacteria are reported in the literature, as the stink bug *Riptortus pedestris* that takes part in a mutualistic symbiosis with members of the *Burkholderia* genus, which are acquired from the soil during the second instar of its development stage. The insect-bacteria association was found in treated fields which showed enrichment for insecticide-degrading *Burkholderia* (Kikuchi et al., 2012). Similar to *R. pedestris*, the intestinal symbiotic bacteria *Citrobacter freundii* enhances the fruit flies’ resistance to trichlorfon insecticide (Cheng et al., 2017).

In addition to containing beneficial microbes, the stink bug intestinal track may also contain pathogens that could be passed to their host plant (Mitchell, 2004). Conducting surveys of the insect intestinal microbiome can help identify possible pathogens, as well as detail what healthy stink bug digestive systems have in common. A study on bacteria associated with *Piezodorus guildinii* included isolating those that can or cannot be transmitted by feeding, *via* the sequencing of PCR products amplified from the V4 region of the 16S rRNA gene (Husseneder et al., 2017). The sequencing of the V4 region of the 16S rRNA gene from *P. guildinii* gut DNA identified 51 putative bacteria species, including several potential plant pathogens. Somewhat similar results were reported in bacteria transmitted by *N. viridula* (Ragsdale et al., 1979).

The unprecedented work presented here focuses on the gut microbiome of two stink bugs that are major pests of soybean, the red-banded stink bug (*Piezodorus guildinii*) collected in Brazil and the United States, and the neotropical brown stink bug (*Euschistus heros*) collected in Brazil. These pests normally colonize pre-flowering soybean and peak infestations are seen at the end of grain filling. They feed by inserting their stylets into the pods, directly reaching the seed (Foster and Daugherty, 1969; Turnipseed and Kogan, 1976; Corrêa-Ferreira et al., 1999; Kimura, 2007). According to Guedes et al. (2012), one stink bug per square meter can cause losses of 125 kg ha^–1^, and losses can reach United States $3.06 billion (CEPEA – USP, 2020; USDA, 2020). Here, we hypothesize that (1) the intestinal microbiome of stink bugs in the United States and Brazil are different, (2) microbiome of stink bugs differ based on ecoregion, and (3) the two stink bug species differ in microbiome composition. To elucidate this hypothesis, we characterized and compared the intestinal microbiome of these two species collected from different locations in Brazil and the United States (Marchesi and Ravel, 2015), and investigated possible microbial families associated with the adaptation of these stink bugs to environments modified by agricultural management.

## Materials and Methods

### Insect Collection and Dissection

In the field, insects were collected in soybean crops in Brazil and the United States and stored in falcon tubes with 70% ethanol at −20°C (Supplementary Table 1). Insects were dissected by removing heads and digestive tracts (including fore-, mid-, and hind guts with attached Malpighian tubes) using sterile forceps and storing them in 1.5 mL microfuge tubes at −20°C until used for DNA extraction.

### DNA Extraction

For bacterial DNA extraction, a modified CTAB method was used as follows. The digestive tracts samples were placed in 1.5 mL microtubes containing 350 μL 2% CTAB extraction buffer (20 mM EDTA, 0.1 M Tris–HCl pH 8.0, 1.4 M NaCl, 2% CTAB, plus 0.4% b-mercaptoethanol added just before use), 10 μL proteinase k, and five stainless steel 2.8 mm beads. An additional 350 μL of 2% CTAB was added per tube and the solution incubated at 65°C for 60 min, and mixed by inversion every 15 min to macerate. Next, 600 μL chloroform-isoamylalcohol (24:1) was added and the tubes were gently mixed for 1 min, followed by centrifugation for 15 min at 10,000 rpm. Immediately after centrifuging, 600 μL of the supernatant of each tube was transferred to a fresh tube with 350 μL cold isopropanol (−20°C). Samples were mixed by inversion and held at −20°C for 60 min, followed by centrifugation at 14,000 rpm for 10 min. After centrifugation, it was possible to visualize the DNA at the bottom of each tube. The supernatants were removed, and the DNA pellets were washed with 1,000 μL of 70% ethanol, centrifuged at 14,000 rpm for 5 min. The ethanol was discarded and 500 μL 100% ethanol was added, centrifuged at 14,000 rpm for 10 min, the ethanol discarded, and the tubes were set to dry for at least 3 min in a sterile cabinet with the tubes inverted over a filter paper at room temperature. The DNA pellets were suspended in 50 μL TE buffer (10 mM Tris–HCl pH 7.6, 1 mM EDTA pH 7.6) plus 2 μL ribonuclease (RNAse 20 mg/mL), incubated at 37°C for 1 h, and stored at −20°C (Doyle and Doyle, 1987).

### PCR Amplification and Sequencing

To fully characterize the microbiome at the *Bacteria* Domain, the PCR primer pair 515F (5′-GTGYCAGCMGCCGCGGTAA) and 806R (5′-GGACTACNVGGGTWTCTAAT) was used for amplifying the V4 region of the bacterial 16S rRNA gene, resulting in an amplicon length of 292 bp. PCR reactions were performed on a high-throughput Fluidigm PCR platform (Biomark HD) at the Roy J. Carver Biotechnology Center, the University of Illinois following the procedure outlined by Muturi et al. (2017). DNA samples were diluted to 2 ng/μL prior to amplification and processed with the Roche High Fidelity Fast Start Kit and 20X Access Array loading reagent according to Fluidigm protocols. To generate final PCR amplicons prepared for subsequent Illumina sequencing, two sets of primers were utilized simultaneously in one reaction. The first primer set had the Fluidigm-specific primers CS1 (5′-ACACTGACGACATGGTTCTACA) and CS2 (5′-TACGGTAGCAGAGACTTGGTCT) added to the 5′ end of all the ribosomal-specific primers mentioned above. The second primer set contained the same Fluidigm-specific primers attached to the Illumina i5 primer (5′-AATGATACGGCGACCACCGAGATCT) and barcoded i7 primer (5′-CAAGCAGAAGACGGCATACGAGAT-XXXXXXXXXX). All primers were synthesized by IDT Corp., (Coralville, IA, United States). The mastermix was aliquoted into 48 wells of a PCR plate. To each well, 1 μL DNA sample and 1 μL Fluidigm Illumina linkers with unique barcodes were added. On a separate plate, primer pairs were prepared and aliquoted. 20X primer solutions were prepared by adding 2 μL of each forward and reverse primer (50 μM), 5 μL of 20X Access Array Loading Reagent, and water to a final volume of 100 μl. The final primer concentration in the reactions was 50 nM each. Samples (4 μL each) were loaded in the sample inlets and 4 μL of primer loaded in primer inlets of a previously primed Fluidigm 48.48 Access Array IFC. The IFC was placed in the Juno microfluidic machine (Fluidigm Corp.) for the loading of all primer/sample combinations, amplification, and harvest. All samples were run on a Fragment Analyzer (Advanced Analytics, Ames, IA, United States), and amplicon regions and expected sizes were confirmed. Samples were then pooled in equal amounts according to product concentrations. The pooled products were size selected on a 2% agarose E-gel (Life Technologies) and extracted from the isolated gel slices with a Qiagen Gel Extraction kit (Qiagen) using a Qiacube robot. Cleaned, size-selected products were run on an Agilent Bioanalyzer to confirm appropriate profiles and determination of average sizes.

PCR amplicons for the 288 samples (286 insects and two water negative control) were sequenced on two MiSeq flow cells of 301 cycles from each end of the fragments using a MiSeq 600-cycle sequencing kit version 3 at the Roy J. Carver Biotechnology Center, University of Illinois. Read length was 300 nt. The resulting fastq files were demultiplexed with the bcl2fastq v2.17.1.14 Conversion Software (Illumina).

### Sequence Data Processing

For 288 sequence libraries of the bacterial 16S rRNA gene V4 region, the software Cutadapt v1.12 was used to trim specific V4 primer sequences on both 5′ and 3′ ends of the reads (Martin, 2011). Since the primers for amplifying this region contained ambiguous positions, the option -match-read-wildcards was set to “on.” Adapter-trimmed paired-end reads were imported into software Pear v0.9.5 (Zhang et al., 2014) that merged sequences into single fragments using overlapping regions to correct sequencing errors and yield higher quality. Reverse complement sequences of merged fragments were created with an in-house script.

The software IM-TORNADO v2.0.3.2 was used to do quality filtering and taxonomy assignment for processed sequences generated by the V4 regions above (Jeraldo et al., 2014). The Trimmomatic program was implemented by IM-TORNADO to do quality trimming, with a hard cutoff of a PHRED score of Q30 at ends of reads (LEADING: 3 and TRAILING: 3), a 4-base average score cutoff of Q15 (SLIDING WINDOW: 4:15) and a minimum read length cutoff of 75% of the original read length. For the taxonomy assignment, reads from the bacterial V4 region were aligned against the Ribosomal Database Project 10 (RDP10) (Cole et al., 2014) and the OTUs with 97% of similarity were clustered. Three BIOM files were generated separately by forward-reads, reverse-reads, and paired-reads for the region, containing the OTUs and counts corresponding to each OTU in each sample; forward-read BIOM files were used for downstream analyses due to the best result. Quality filtering was performed to remove low-quality OTUs and samples using QIIME-v1.9.1 (Caporaso et al., 2010).

The sufficiency of sequencing coverage was evaluated by alpha diversity and rarefaction analyses. Three metrics were used to estimate the alpha diversity of the microbiome among all the stink bug samples, i.e., the number of observed OTUs, Chao1, and Shannon.

The datasets generated for this study can be found in the NCBI Sequence Read Archive under the identification PRJNA764175 (*Euschistus heros*) and PRJNA764176 (*Piezodorus guildinii*).

### Data Analysis

To compare the bacterial community structure and composition among the samples the data was rarefied and we used the Non-metric Multidimensional Scaling (NMDS) using Bray-Curtis as a distance metric, and the Phyloseq (McMurdie and Holmes, 2013) package in R. To test whether the samples harbored significantly different bacterial community compositions, permutational multivariate analysis of variance (PERMANOVA) (Anderson, 2001) was performed.

Diversity measurements were calculated using the Phyloseq package in R and included alpha diversity (Shannon index) and richness (number of observed OTUs, and Chao1 richness estimator). The Kruskal–Wallis non-parametric test was used to compare multiple groups and the pairwise Wilcoxon non-parametric test was used to compare samples grouped in ecoregions. Further, to determine the differential abundance of the top ten bacterial families between the ecoregions, we performed the Wilcoxon non-parametric parameter test for each bacterial family used.

Recent discoveries indicated that *E. heros* from the north and the south of Brazil were composed of two distinguished lineages. With the expansion of soybean in the Brazilian Cerrado, these two lineages could meet and produce hybrids more adapted to the climates and even to new hosts, such as cotton (Soares et al., 2018; Zucchi et al., 2019b). Thus, we performed analyses to investigate the relationships between the stink bug intestinal microbiomes and the proposed lineages of *E. heros*: north (Teresina/PI, Palmeirante/TO), south (Piracicaba/SP, Anhembi/SP, and Ponta Grossa/PR), and hybrids (the other localities).

## Results

### Sequences and Samples Quality Control

For the 286 stink bug PCR samples of the bacterial 16S rRNA V4 sequence, the Illumina MiSeq sequencing produced 7.5 million reads in one lane, and the mean number of raw reads ranges from about 7,000–53,000 per sample for different locations. Reads filtering procedures including merging reads and trimming low-quality bases removed large percentages of reads from most locations, with 33–73% reads remaining, but the stringency of filtering left high-quality sequence data for subsequent analyses (Supplementary Table 2). After OTU identification, low-quality samples were filtered out for downstream analysis, leaving a total of 192 samples out of 286, with 75 samples from *E. heros* collected from 10 sites in Brazil and 117 samples from *P. guildinii* collected from three sites in Brazil and four sites in the United States (Supplementary Table 2).

The depth of sequencing was sufficient as shown by *P. guildinii* rarefaction plot that began to plateau by 1,000–1,500 reads per sample (Supplementary Figure 1B). Similarly, the *E. heros* rarefaction plot also began to plateau by 1,000–1,500 reads per sample for most locations, although the Teresina and Uberlândia locations might have benefited from additional sequencing as those plotlines still had a slightly positive slope at 1,500 (Supplementary Figure 1A). To accomplish this study we used a total of 2,141 OTUs.

### Microbiome Characterization of *Euschistus heros*

**FIGURE 1:**
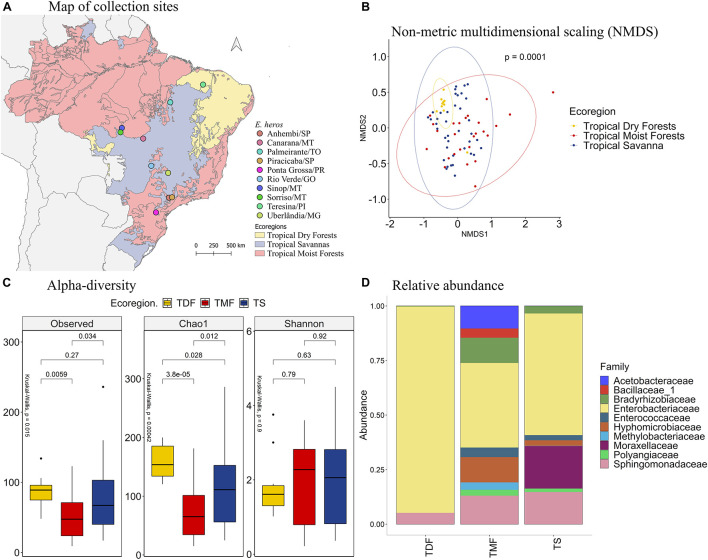
**(A)** Map of collection sites of *E. heros*, classified by ecoregion in Brazil. **(B)** NMDS of Bray-Curtis similarity matrix among 75 samples from the intestinal microbiome of *E. heros*, for the different ecoregions from Brazil. The ellipses are showing clustering between ecoregions. Permutational multivariate analysis of variance (PERMANOVA) is indicated in the upper right. **(C)** Alpha diversity levels based on Observed OTU, Chao1 index and Shannon index for the different ecoregions. The non-parametric test (Kruskal–Wallis) is indicated in the upper left of each graph. Pairwise comparison (Wilcoxon) is indicated between Ecoregions. **(D)** Analysis of the ten most abundant bacteria families in the intestinal microbiome of *E. heros* collected in Brazil, classified by ecoregion.

Comparing the different collection sites of *E. heros* using the non-metric multidimensional scaling (NMDS) analysis, the bacterial communities present in the gut of these stink bugs clustered according to the following ecoregions of Brazil: tropical dry forests (TDF), tropical moist forests (TMF), and tropical savanna (TS) (Figures 1A,B, PERMANOVA *P* = 0.0001). Therefore, subsequent analyses were based on these ecoregions.

The species richness, based on the number of observed OTUs, is different among the ecoregions (*p* = 0.015). *E. heros* collected in tropical dry forests (*p* = 0.0059) and tropical savanna (*p* = 0.034) had significantly higher observed OTUs than those collected in tropical moist forests. This result was confirmed by the richness estimator Chao1 (*p* = 0.0004). *E. heros* collected in sites from tropical dry forests (*p* = 3.8e-05) and Tropical Savanna (*p* = 0.012) had more richness of species than *E. heros* collected in tropical moist forests. However, Shannon’s diversity, an index that takes into account both abundance and evenness of the species present, did not show significant differences among ecoregions. A noticeable observation would be that the *E. heros* microbiome from tropical dry forest showed the highest richness of species (OTUs and Chao1) but the lowest Shannon diversity index, indicative of low evenness/equitability of species within this bacterial community (Figure 1C).

The most abundant bacterial families in the intestinal microbiome of *E. heros* were Acetobacteraceae, Bacillaceae, Bradyrhizobiaceae, Enterobacteriaceae, Enterococcaceae, Hyphomicrobiaceae, Methylobacteriaceae, Moraxellaceae, Polyangiaceae, and Sphingomonadaceae. The Acetobacteraceae and Bacillaceae were unique to insects sampled from the tropical moist forests (Figure 1D).

Comparing the bacterial families across ecoregions, stink bugs from the tropical moist forests had a significantly higher mean proportion of the Bradyrhizobiaceae than dry forests (*p* = 0.012) and savanna (*p* = 0.016) (Supplementary Figure 2). Although Enterobacteriaceae was the most abundant family in stink bugs from all three ecoregions (Figure 1D), it was more abundant in the tropical dry forests, followed by the tropical savanna and moist forests (Supplementary Figure 2). The Moraxellaceae only existed abundantly in stink bugs from tropical savanna (∼20%) whereas in the other two ecoregions they were not detected, or present at a very low level.

**FIGURE 2:**
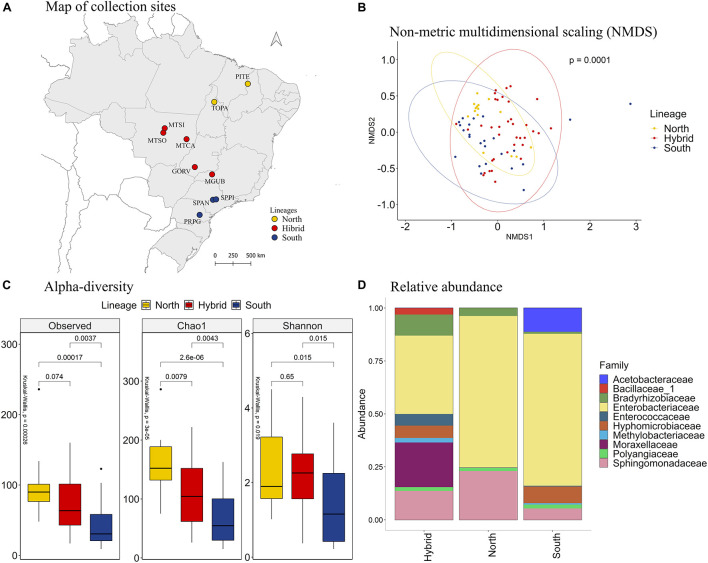
**(A)** Map of collection sites of *E. heros*, classified by lineages in Brazil. **(B)** NMDS of Bray-Curtis similarity matrix among 75 samples from the intestinal microbiome of *E. heros*, for the different lineages. The ellipses are showing clustering between lineages. Permutational multivariate analysis of variance (PERMANOVA) is indicated in the upper right. **(C)** Alpha diversity levels based on Observed OTU, Chao1 index and Shannon index for the different lineages. The non-parametric test (Kruskal–Wallis) is indicated in the upper left of each graph. Pairwise comparison (Wilcoxon) is indicated between lineages. **(D)** Analysis of the ten most abundant bacteria families in the intestinal microbiome of *E. heros* collected in Brazil, classified by lineages.

Comparing the different lineages, the NMDS analysis showed that the bacterial species present in our samples clustered significantly by stink bug lineages (Figures 2A,B, PERMANOVA *p* = 0.0001). The species richness, based on the observed OTU and Chao1 index, is higher in samples from the north lineage than the south lineage, and the hybrids have an intermediate species richness. The species diversity, based on the Shannon index, is bigger on the north and hybrid lineages than the south lineage of *E. heros* (Figure 2C).

Analyzing the relative abundance of the 10 most abundant bacterial families in each lineage, we can see that the hybrid lineages had more bacterial families than the north and south lineages and the proportion of these families present in the gut of the hybrids lineages are more evenly distributed than in pure lineages (Figure 2D). Furthermore, the family Bacillaceae is unique in the hybrids lineages and the Acetobacteraceae family is unique in the south lineage.

Comparing the bacteria families among lineages, the family Bradyrhizobiaceae was the most abundant in *E. heros* from hybrids lineages than either of the pure lineages. The family Hyphomicrobiacea was the most abundant in *E. heros* from hybrid lineages than north lineages. The families Moraxellaceae and Sphingomonadaceas were the most abundant in hybrid lineages than south lineages and the Enterobacteriaceae family was the most abundant in both pure lineages than hybrids (Supplementary Figure 3).

### Microbiome Characterization of *Piezodorus guildinii*

**FIGURE 3:**
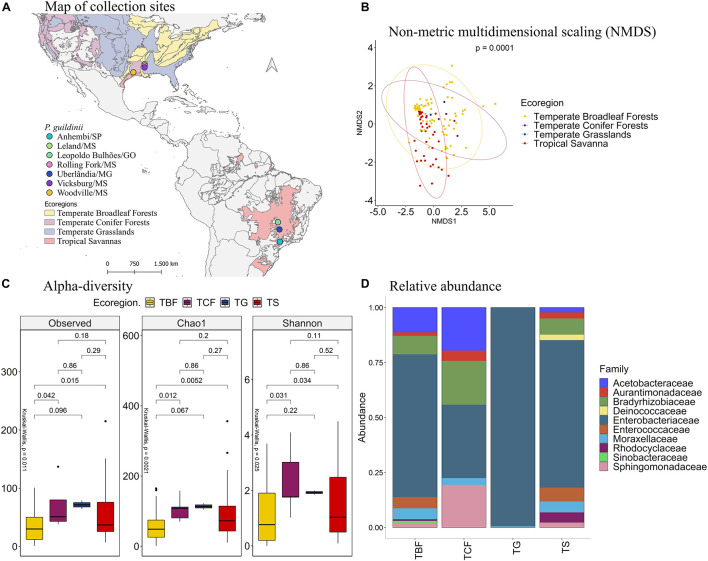
**(A)** Mapping of collection sites of *P. guildinii*, classified by ecoregion in Brazil and the United States. **(B)** NMDS of Bray-Curtis similarity matrix among 117 samples from the intestinal microbiome of *P. guildinii*, for the different ecoregions from Brazil and United States. The ellipses are showing clustering between ecoregions. Permutational multivariate analysis of variance (PERMANOVA) is indicated in the upper right. **(C)** Alpha diversity levels based on Observed OTU, Chao1 index and Shannon index for the different ecoregion. The non-parametric test (Kruskal–Wallis) is indicated in the upper left of each graph. Pairwise comparison (Wilcoxon) is indicated between Ecoregions. **(D)** Analysis of the ten most abundant bacteria families in the intestinal microbiome of *P. guildinii* collected in Brazil and the United States, classified by ecoregion.

The comparison of the different collection sites of *P. guildinii* by NMDS analysis (Figure 3B) revealed that the bacterial species present in the gut of these stink bugs also clustered (*p* of 0.0001) according to the ecoregions of Brazil and the United States: temperate broadleaf forests (TBF), temperate conifer forests (TCF), temperate grasslands (TG), and tropical savanna (TS) (Figure 3A). Therefore, subsequent analyses were based on these ecoregions.

The bacterial species diversity across ecoregions was significantly different based on the alpha diversity index, the number of OTUs, and Chao1 index (*p* = 0.0072 and *p* = 0.0035, respectively) (Figure 3C). On average, *P. guildinii* collected in temperate grasslands had more bacterial species than *P. guildinii* collected in the other ecoregions. Considering the richness estimator Chao1, the values were also significantly different (*p* = 0.0035). *P. guildinii* from tropical savanna were richer in species than insects from temperate broadleaf forests (*p* = 0.0025). Shannon’s diversity showed a significant difference among ecoregions (*p* = 0.018). *P. guildinii* from temperate conifer forests (*p* = 0.015) and tropical savanna (*p* = 0.044) had higher equitability than temperate broadleaf forests, confirming the lowest species diversity from insects of temperate broadleaf forests at Shannon index (Figure 3C).

When focusing on the microbial abundance of *P. guildinni* at the family level, the most abundant bacterial families were Acetobacteraceae, Aurantimonadaceae, Bradyrhizobiaceae, Deinococcaceae, Enterobacteriaceae, Enterococcaceae, Moraxellaceae, Rhodocyclaceae, Sinobacteraceae, and Sphingomonadaceae. Among these families, the Sinobacteraceae family was found only in insects from temperate broadleaf forests and it existed at a low proportion. The Rhodocyclaceae family was found only in insects from this ecoregion and temperate broadleaf forests in low proportion (Figure 3D).

Comparing the bacterial families present in the intestine of *P. guildinii* insects shared by more than one ecoregions, the Acetobacteraceae family was significantly more abundant in insect guts from temperate broadleaf forests than insect guts from tropical savanna (*p* = 0.044). The Aurantimonadaceae was more abundant in *P. guildinii* from temperate conifer forests than temperate broadleaf forest and tropical savanna (*p* = 0.015 and 0.029). The Bradyrhidobiaceae family was more abundant in *P. guildinii* from temperate broadleaf forests than tropical savanna. Lastly, the Enterobacteriaceae family, as a major component in all four ecoregions, exhibited a lower proportion in temperate conifer forests than the other three ecoregions, but the difference did not reach statistical significance either, due to large variation (Supplementary Figure 4).

**FIGURE 4:**
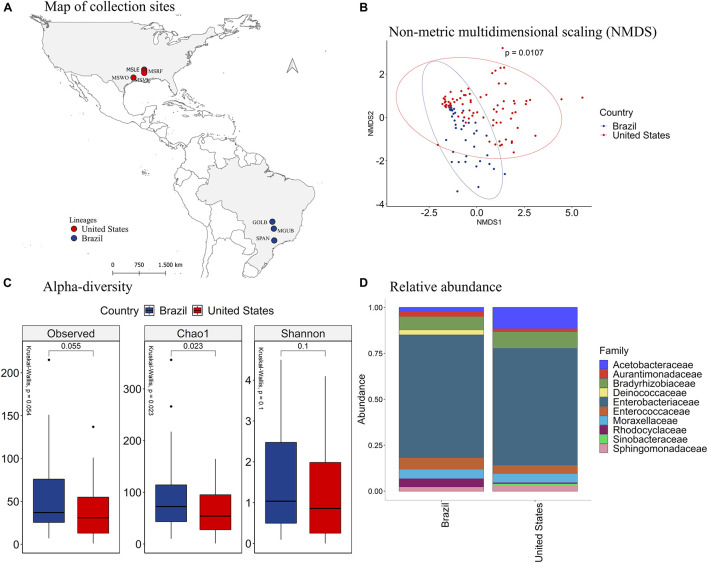
**(A)** Mapping of collection sites of *P. guildinii*, classified by Country in Brazil and the United States **(B)** NMDS of Bray-Curtis similarity matrix among 117 samples from the intestinal microbiome of *P. guildinii* for the different countries, Brazil and United States. The ellipses are showing clustering between countries. Multivariate analysis of variance (PERMANOVA) is indicated in the upper right. **(C)** Alpha diversity levels based on Observed OTU, Chao1 index and Shannon index for the different countries. The non-parametric test (Kruskal–Wallis) is indicated in the upper left of each graph. Pairwise comparison (Wilcoxon) is indicated between countries. **(D)** Analysis of the ten most abundant bacteria families in the intestinal microbiome of *P. guildinii* collected in Brazil and the United States, classified by country.

Since the species *P. guildinii* co-occurred in both Brazil and the United States, we further explored the association between its intestinal microbiome and the country of origin (Figure 4A). The NMDS analysis (Figure 4B) showed distinct groups of bacterial species in insects from Brazil and the United States (*p* = 0.0107). According to the number of observed OTU (*p* = 0.015) and the richness estimator Chao1 (*p* = 0.0087), *P. guildinii* sampled from Brazil were richer in intestinal bacterial species than insects from the United States (Figure 4C).

The Deinococcaceae family occurred only in stink bugs collected from Brazil, and the Sinobacteraceae occurred only in insects collected from the United States (Figure 4D). The families Acetobacteraceae, Bradyrhizobiaceae, and Moraxellaceae were significantly more abundant in *P. guildinii* from the United States than Brazil, and the family Rhodocyclaceae was more abundant from Brazilian insects (Supplementary Figure 5). Enterobacteriaceae, the most abundant bacterial family in both countries, constituted about 80% of the gut microbiome of both countries and showed no significant difference (Figure 4D and Supplementary Figure 5).

### Analysis of *Euschistus heros* and *Piezodorus guildinii* Combined

**FIGURE 5:**
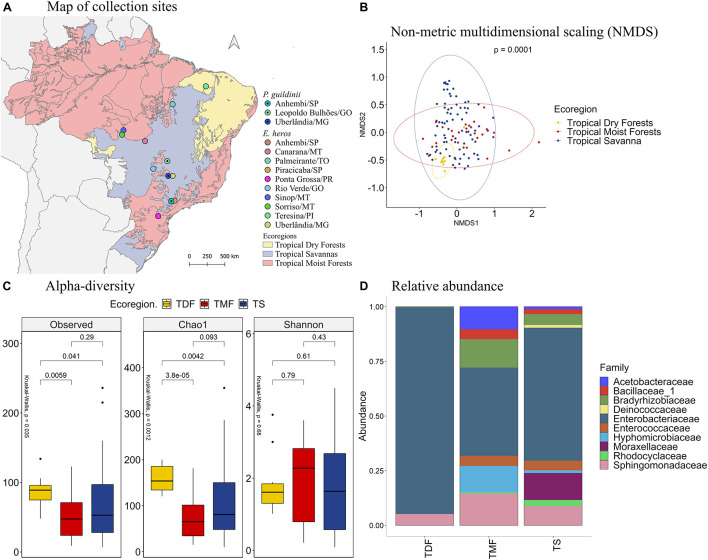
**(A)** Mapping of collection sites of *E. heros* and *P. guildinii*, classified by ecoregion in Brazil. **(B)** NMDS of Bray-Curtis similarity matrix among 115 samples from the intestinal microbiome of *E. heros* and *P. guildinii*, for the different ecoregion from Brazil. The ellipses are showing clustering between ecoregion. Multivariate analysis of variance (PERMANOVA) is indicated in the upper right. **(C)** Alpha diversity levels based on Observed OTU, Chao1 index and Shannon index for the different ecoregion. The non-parametric test (Kruskal–Wallis) is indicated in the upper left of each graph. Pairwise comparison (Wilcoxon) is indicated between Ecoregions. **(D)** Analysis of the ten most abundant bacteria families in the intestinal microbiome of *E. heros* and *P. guildinii* collected in Brazil, classified by ecoregion.

We further combined the microbiome data of *E. heros* and *P. guildinii* and clustered samples by ecoregions (Figure 5A). With PERMANOVA, the bacterial species present in stink bugs displayed distinct compositions by different ecoregions with a significant *p* at 0.0001 (Figure 5B). As seen in the NMDS analysis (Figure 5B), samples from tropical dry forests grouped and were separated from samples from tropical moist forests and tropical savanna. Therefore, subsequent analyzes were based on ecoregions.

According to the number of observed OTUs, we found that stink bugs from tropical dry forests contained more abundant bacterial species than stink bugs from the tropical moist forests (*p* = 0.0059) and tropical savanna (*p* = 0.041). The same occurs when the richness estimator Chao1 is compared (*p* = 3.8e-05 and *p* = 0.0042, respectively). Lastly, Shannon’s index showed that the species diversity of all three ecoregions did not differ significantly from each other (Figure 5C).

The most abundant bacterial families present in the digestive tracts of *E. heros* and *P. guildinii* were Acetobacteraceae, Bacillaceae, Bradyrhizobiaceae, Deinococcaceae, Enterobacteriaceae, Enterococcaceae, Hyphomicrobiaceae, Moraxellaceae, Rhodocyclaceae, and Sphingomonadaceae, with Enterobacteriaceae comprising more than 80% of the bacterial community. The families Deinococcaceae and Moraxellaceae only existed in the tropical savanna group (Figure 5D). Comparing the bacterial families present in two or more ecoregions, the Bradyrhizobiaceae family was significantly more abundant in insects from tropical moist forests than tropical dry forests and tropical savanna. The Enterobacteriaceae family was most abundant in insects from tropical dry forests, followed by insects from tropical savanna (Supplementary Figure 6).

**FIGURE 6:**
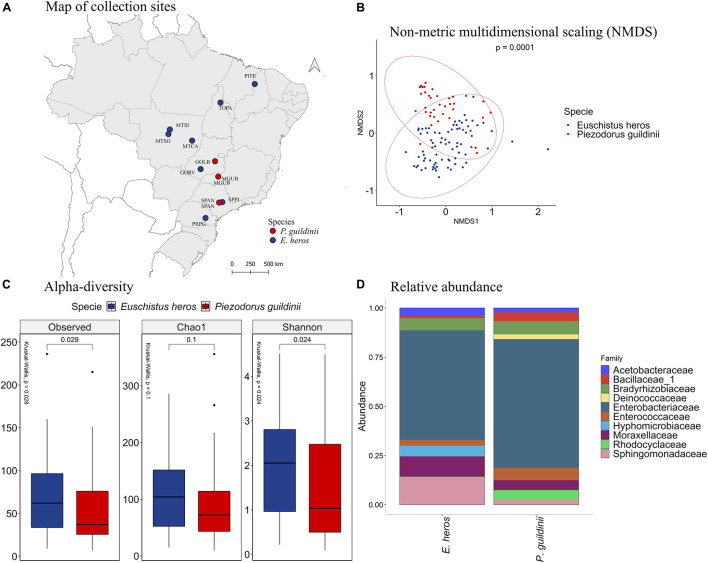
**(A)** Mapping of collection sites of *E. heros* and *P. guildinii*, classified by species in Brazil. **(B)** NMDS of Bray-Curtis similarity matrix among 115 samples from the intestinal microbiome of *E. heros* and *P. guildinii* collected in Brazil. The ellipses are showing clustering between species. Multivariate analysis of variance (PERMANOVA) is indicated in the upper right. **(C)** Alpha diversity levels based on Observed OTU, Chao1 index and Shannon index for the different species. The non-parametric test (Kruskal–Wallis) is indicated in the upper left of each graph. Pairwise comparison (Wilcoxon) is indicated between Ecoregions. **(D)** Analysis of the ten most abundant bacteria families in the intestinal microbiome of *E. heros* and *P. guildinii* collected in Brazil, classified by species.

Comparing the species of stink bugs between *E. heros* and *P. guildinii* (Figure 6A), the NMDS analysis showed that these pentatomids had different bacterial species in their intestines, which can be separated into two groups with a *p* = 0.0001 (Figure 6B). Analyzing the diversity indexes, the number of observed OTUs showed that *E. heros* had more bacterial species than *P. guildinii*. Based on Shannon’s index, the bacterial community in the gut of *E. heros* had significantly higher diversity than *P. guildinii*, which also suggests a relatively even distribution of species in the *E. heros* intestines (Figure 6C).

The composition of bacterial communities in the two stink bugs was similar at the family level (Figure 6D). Enterobacteriaceae was the major family and constitute more than 50% of the bacterial species in both stink bugs. The two other major bacterial families shared by the two stink bug species were Bradyrhizobiaceae and Moraxellaceae. However, differences can be observed as well. Hyphomicrobiaceae occurred only in *E. heros* and Deinococcaceae occurred only in *P. guildinii* (Supplementary Figure 7).

**FIGURE 7:**
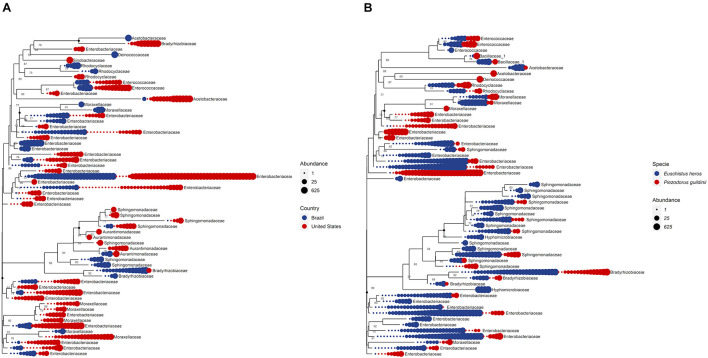
**(A)** Phylogenetic tree comparing top 10 bacteria abundance at family level among countries. **(B)** Phylogenetic tree comparing top 10 bacteria abundance at family level among species.

The phylogenetic relationship of the OTUs from the top 10 most abundant bacterial families was identified to understand their distribution (Figure 7). Analyzing the OTUs among *P. guildinii* from Brazil and the United States, 13 unique OTUs belonged only to Brazil and 19 unique OTUs belonged only to the United States. Most OTUs were not related among countries. The families Enterococcaceae (2 OTUs), Acetobacteraceae (1 OTU), Enterobacteriaceae (15 OTUs), Aurantimonadaceae (1 OTU), and Bradyrhizobiaceae (1 OTU) belonged to *P. guildinii* from both countries. This difference may be related to bacterial diversity among environments. On the other hand, comparing the OTU from the ten most abundant bacterial families among *E. heros* and *P. guildinii* from Brazil, 16 unique OTUs belong only to *E. heros* and seven unique OTUs belong only to *P. guildinii.* The families Enterococcaceae (2 OTUs), Bacillaceae (2 OTUs), Acetobacteraceae (1 OTU), Rhodocyclaceae (2 OTUs), Moraxellaceae (3 OTUs), Enterobacteriaceae (12 OTUs), Sphingomonadaceae (7 OTUs), and Bradyrhizobiaceae (3 OTUs) belonged to both stink bug species. This difference is most interesting, *E. heros* had twice the number of unique bacteria than *P. guildinii*, which may be favoring the adaptation of this species over the other since it is the most difficult to control today (Figure 7).

## Discussion

This study evaluated the insect intestinal microbiome, seeking to describe the structure and composition among different regions. Our results revealed a higher bacterial species richness in the stink bug intestinal tract from dry regions in *E. heros* and temperate grasslands in *P. guildinii*. Comparing the *P. guildinii* intestinal microbiome in both countries, we observed a higher bacterial species richness in stink bugs collected in Brazil than in the United States. On the other hand, comparing both species in Brazil, *E. heros* was richer in bacterial species than *P. guildinii*.

### Microbiome Structure and Diversity

Differences in bacterial species richness found in *E. heros* and *P. guildinii* from different ecoregions, lineages, and species can be mainly explained by species behavioral, environmental, and genetic factors. *E. heros* and *P. guildinii* can feed on different food sources, including native and weed plants, this pattern allows individuals to frequently transfer to various areas throughout their development (Panizzi et al., 2012). According to Engel and Moran (2013), the microorganisms present in the insect gut can be acquired in each generation from the outside environment, such as soil. Studies that evaluated the diversity and biogeography of soil bacterial communities found that bacterial diversity varied across the ecosystem types, e.g., dry forest and dry grassland soils were more diverse than humid temperate forest and tropical forests (Fierer and Jackson, 2006). Our results showed that *E. heros* had higher species richness in tropical dry forests than other ecoregions, which might support the previous finding that the intestinal bacterial diversity could be affected by environmental factors.

Once acquired from the environment, the microorganism goes through a series of physicochemical conditions of gut compartments, such as pH, redox potential, and availability of particular substrates, that can be selective for particular species (Engel and Moran, 2013). In many insects, gut bacterial communities vary among individuals within a species and appear to consist largely of bacteria not specifically adapted to living in the guts of their host species (Engel and Moran, 2013). Corroborating our study, Priya et al. (2012) found highly variable gut communities in field-collected *Helicoverpa armigera* from different locations with the influence of the host plant.

The indiscriminate and large-scale use of phytosanitary products in soy production regions in Brazil may affect the soil microbiome and even insect’s gut microbiota, which may be the main explanation for the grater diversity of bacteria in dry tropical forests.

(Spehar, 1995). A previous study on the gut microbiota composition of honey bees indicated that the neonicotinoids pesticides exposition may influence dominant honey bee gut bacteria (Jones et al., 2018). As stated earlier, stink bugs can feed on native plants and Tropical dry forests, due to the less intensive cultivation of soybeans than other ecoregions, may have a greater diversity of native plants to feeding.

Analyzing the species *E. heros* separated by northern and southern lineages, northern lineage individuals present higher species richness, followed by individuals of the hybrid and southern lineage. An explanation would be that individuals of the northern lineage (specialized in cotton) have genetic characteristics that when expressed allow the establishment of a greater number of bacterial species when compared to the southern lineage (specialized in soy) (Soares et al., 2018; Zucchi et al., 2019b). Thus, the hybrids of these two lineages present intermediate species richness.

It is important to note that the insect’s lineage and the ecoregion to which it belongs can be equally important to the difference in the microbial community, as evidenced by the PERMANOVA.

We found that *P. guildinii* displayed a higher intestinal bacterial diversity from Brazil than the United States, which might be mainly explained by genetic and environmental factors. For genetic factors, Zucchi et al. (2019a) evidenced distinct *P. guildinii* lineages from Brazil and the United States using Genotype-By-Sequencing. These genetic differences in stink bug lineages might account for the differences in the intestinal microbiome changing the physicochemical conditions of the intestine and, consequently, the microbial composition of the different lineages. For environmental factors, the discrepancy of climatic conditions of each country could be relevant because the performance of seed-sucking heteropterans is affected by a variety of abiotic factors. The high temperature could increase food ingestion and, consequently, the ingestion of bacteria (Slansky-Júnior and Panizzi, 1987).

Tropical areas feature broad offerings of resources to bacteria species due to a higher diversity of environment and microclimates. Other pentatomids such as *E. heros*, enter in reproductive diapause under a photoperiod of 12 h or less, reducing feeding activity (Mourão and Panizzi, 2002). Collections in Brazil were made at latitudes below 25°, while in the United States they were made at latitudes above 30°. This difference means that in winter Brazil has more hours of light than the United States, possibly resulting in greater food activity of stink bugs in Brazil and thus affecting the diversity of the intestinal microbiota. The bacterial diversity found in the gut of insects in different ecoregions of the country and both countries is possibly related to the microbial diversity of the environment in which this insect is inserted.

### Microbiome Composition

Bacterial abundance may be related to the stink bug environment and eating habits. Previous studies of insect gut symbionts have shown that diet change shifts relative abundances of bacteria in the insect gut rather quickly (Husseneder et al., 2009). Besides that, within the insect, symbiotic microorganisms have to face the host’s innate immune system (Gross et al., 2009).

A vast majority of microbes constituting the gut microbiota are referred to as commensal microbes. By definition, commensals gain benefits from the host but are neither beneficial nor harmful to the host. However, commensals can be viewed as a pool of gut-interacting microbes with potential regulatory functions in host physiology (Lee et al., 2017).

The Acetobacteraceae family was found in both species of stink bugs. However, they were the majority in insect guts collected in the tropical moist forests and temperate broadleaf and mixed forests. Members of this family are symbionts of a wide variety of insects, providing nutrition to insects on limited sugar-rich diets (Crotti et al., 2010) and are commonly found in the guts of honey bee (Corby-Harris et al., 2014). These locations have less intensive soybean cultivation than other collection sites, allowing these insects to feed on less nutrient-rich crops. Possibly these bacteria are providing nutrients necessary for the survival of these insects in this environment.

The family Bacillaceae was found only in *E. heros* individuals collected in tropical moist forests. As discussed earlier, this ecoregion has less intensive cultivation of soybean, forcing this species to remove nutrients from the cultural remains between two cycles (Panizzi et al., 2012). So, the Bacillaceae family has a fundamental role in the survival of this stink bug, as many *Bacillus* isolates have the ability to break down cellulose, hemicellulose, and pectin. They are saprophytes that participate in the carbon, nitrogen, sulfur, and phosphorus cycles in natural habitats (Mandic-Mulec et al., 2015), enabling the acquisition of nutrients from these previously inaccessible cells. According to Husseneder et al. (2017), the genus *Bacillus* is the second major genus transmitted to the plant by *P. guildinii* feeding. Species belonging to this genus is known as a promoter of plant growth, biocontrol agent, and crop protective inhibit fungal and bacterial pathogens on seedlings. Bacteria belonging to the γ-Proteobacteria class, as Enterobacteriaceae and Moraxellaceae, supplement the nutritionally poor diet of phloem sap through the provision of essential amino acids (Douglas, 2006).

The Moraxellaceae family was found in both stink bug species. *E. heros* had this family only in individuals collected in the tropical savanna, known as the Cerrado. However, this family was found in *P. guildinii* in all ecoregions. This family can have an important function for these insects to acquire new hosts such as cotton. According to Zucchi et al. (2019b), hybrids lineages of *E. heros* are colonizing the cotton crop in Cerrado due to its improvement of the detoxification capability acquired of existing adaptations from recombination of genes from northern populations (specialized in cotton) with southern populations (specialized in soybean). It is interesting because this family was found in all sites where the use of agrochemicals is high. Moreover, some Acinetobacter species are supposedly aiding herbivores in overcoming plant defenses through detoxification of phytochemicals (Mason et al., 2014). Furthermore, some bacteria of the Acinetobacter genus can be transmitted by the red-banded stink bug feeding and have potentially beneficial effects on insects (Husseneder et al., 2017).

The Enterobacteriaceae was the most abundant family in both stink bugs. Corroborating our study, Enterobacteriaceae was found in *Spodoptera frugiperda* as the most abundant family (Rolim, 2014). Many species can exist as free-living in diverse ecological niches, both terrestrial and aquatic environments, and some are associated with animals, plants, or insects only (Teixeira and Merquior, 2014). Besides that, the genus *Erwinia* of the family Enterobacteriaceae contains mostly plant pathogens. *Erwinia persicina*, one of the major bacteria species found in the guts of red-banded stink bug, is a pathogen of legume plants (Husseneder et al., 2017). Enterobacteriaceae was found mostly in insect guts from the tropical dry forest and tropical savanna. Endosymbionts from this family may be performing some functions assisted in adapting these insects to new hosts.

The Rhodocyclaceae family was found mostly in stink bugs from Brazilian tropical savanna. Representatives of this family have been isolated from diverse environments have considerable potential for biodegradation of organic waste material and bioremediation of polluted environments (Rosenberg et al., 2014). So, members of this family may be associated with insecticides degradation and consequently better adaptations of these stink bugs.

## Concluding Remarks

In this work, we assessed the microbiome of the red-banded stink bug *Piezodorus guildinii* collected in Brazil and the United States, as well as the neotropical brown stink bug *Euschistus heros* collected in Brazil. Our analysis showed that the diversity and abundance of intestinal bacteria vary among the two species, ecoregions, and countries within the same species. This variability allows individuals to perform different functions to better adapt to the environments in which they live. The capacity of endosymbionts bacteria for nutrient supply, cellulose breakdown, phytochemical detoxification, and degradation of organic compounds suggested by the analysis is directly related to adaptability, survival, and control difficulty in the field of these stink bugs. As Brazil and the United States are countries with great environmental diversity, we cannot generalize the control method. To find a more sustainable way to control stink bugs, we need to understand the relationship and dependence on intestinal bacteria, and this relationship must be better explored in future studies.

## Data Availability Statement

The datasets presented in this study can be found in online repositories. The names of the repository/repositories and accession number(s) can be found below: https://www.ncbi.nlm.nih.gov/bioproject/PRJNA764175 and https://www.ncbi.nlm.nih.gov/bioproject/?term=PRJNA764176.

## Author Contributions

MM: conceptualization, data analysis, original draft writing, and review and editing. XW: investigation methodology, data analysis, and review and editing. WW and LM: data analysis and review and editing. KA and JP: insect collection and review and editing. SC: funding, conceptualization, and review and editing. MZ: funding, conceptualization, insect collection, and review and editing. All authors contributed to the article and approved the submitted version.

## Conflict of Interest

The authors declare that the research was conducted in the absence of any commercial or financial relationships that could be construed as a potential conflict of interest.

## Publisher’s Note

All claims expressed in this article are solely those of the authors and do not necessarily represent those of their affiliated organizations, or those of the publisher, the editors and the reviewers. Any product that may be evaluated in this article, or claim that may be made by its manufacturer, is not guaranteed or endorsed by the publisher.
