# Supplementary material for: Characterization and Comparison of Intestinal Bacterial Microbiomes of Euschistus heros and Piezodorus guildinii Collected in Brazil and the United States
Source: Front Microbiol. 2021 Oct 27;12:769965. doi: 10.3389/fmicb.2021.769965 (PMC8579040; doi:10.3389/fmicb.2021.769965)
Supplement: Supplementary file 1 [file Data_Sheet_1.pdf]

Characterization and comparison of intestinal bacterial microbiomes of *Euschistus heros* and *Piezodorus guildinii* collected in Brazil and the United States

Matheus Sartori Moro[1,2,Ψ,*], Xing Wu[3,Ψ], Wei Wei[3], Lucas William Mendes[4], Kerry Clint Allen[5], José Baldin Pinheiro[6], Steven John Clough[3,7], Maria Imaculada Zucchi[2,8*]

Supplementary material

Table 1. Collected samples.

Species	Location	Latitude	Longitude
Euschistus heros	Anhembi/SP - BR	-22.789	-48.128
	Canarana/MT - BR	-13.539	-52.164
	Palmeirante/TO - BR	-7.8615	-47.927
	Piracicaba/SP - BR	-22.725	-47.648
	Ponta Grossa/PR - BR	-25.095	-50.163
	Rio Verde/GO - BR	-17.797	-50.900
	Sinop/MG - BR	-11.873	-55.498
	Sorriso/MG - BR	-12.559	-55.714
	Teresina/PI - BR	-5.0892	-42.802
	Uberlândia/MG - BR	-18.911	-48.262
Piezodorus guildinii	Anhembi/SP - BR	-22.789	-48.128
	Leland/MS - USA	33.404	-90.891
	Leopoldo Bulhões/GO - BR	-16.615	-48.740
	Rolling Fork/MS - USA	32.907	-90.877
	Uberlandia/MG - BR	-18.911	-48.262
	Vicksburg/MS - USA	32.317	-90.887
	Washinton C./MS - USA	38.277	-89.350
	Woodville/MS - USA	31.069	-91.308

Table 2. Quality control of reads and samples.

Species	Location	Sample number before filtering	Samples number after filtering	Mean number of raw reads	Mean number of reads after filtering	Mean percentage after filtering
Euschistus heros	Anhembi/SP - BR	10	6	39,695	28,848	70%
	Canarana/MT - BR	10	9	27,976	18,604	69%
	Palmeirante/TO - BR	10	8	8,858	4,659	49%
	Piracicaba/SP - BR	21	12	48,362	38,416	59%
	Ponta Grossa/PR - BR	10	6	7,973	4,406	34%
	Rio Verde/GO - BR	7	4	5,725	2,763	49%
	Sinop/MG - BR	10	9	7,302	3,301	37%
	Sorriso/MG - BR	10	9	14,740	9,587	52%
	Teresina/PI - BR	10	8	53,929	40,878	72%
	Uberlândia/MG - BR	9	7	31,136	22,111	73%
Piezodorus guildinii	Anhembi/SP - BR	29	20	33,507	24,751	66%
	Leland/MS - USA	17	17	13,727	10,139	59%
	Leopoldo Bulhões/GO - BR	14	12	21,221	14,279	55%
	Rolling Fork/MS - USA	37	33	19,117	15,708	60%
	Uberlandia/MG - BR	6	2	8,611	4,181	34%
	Vicksburg/MS - USA	4	0	3,456	1,481	33%
	Washinton C./MS - USA	61	38	36,963	33,413	60%
	Woodville/MS - USA	11	11	7,347	4,365	43%
	**Total**	286	192	7,551,148	4,157,241	

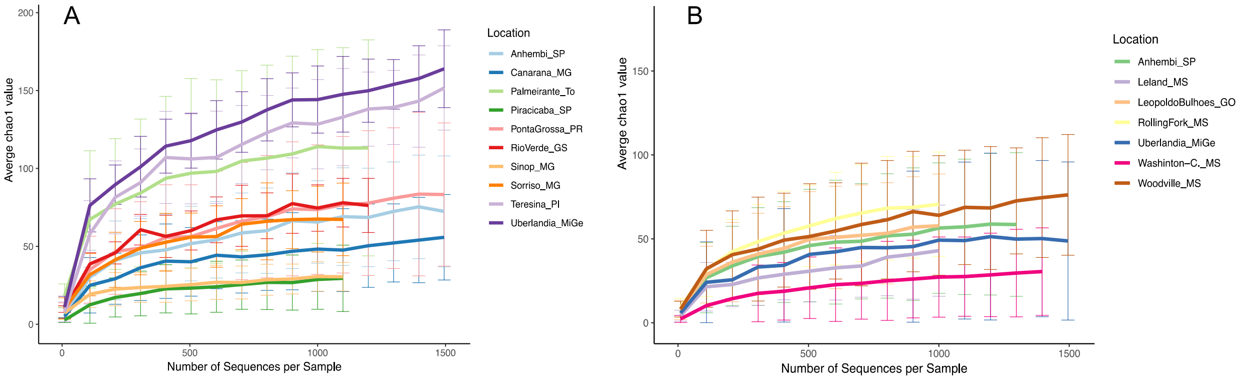

Figure 1. Rarefaction analyses using chao1 index of alpha diversity for *E. heros* (A) and *P. guildinii* (B).

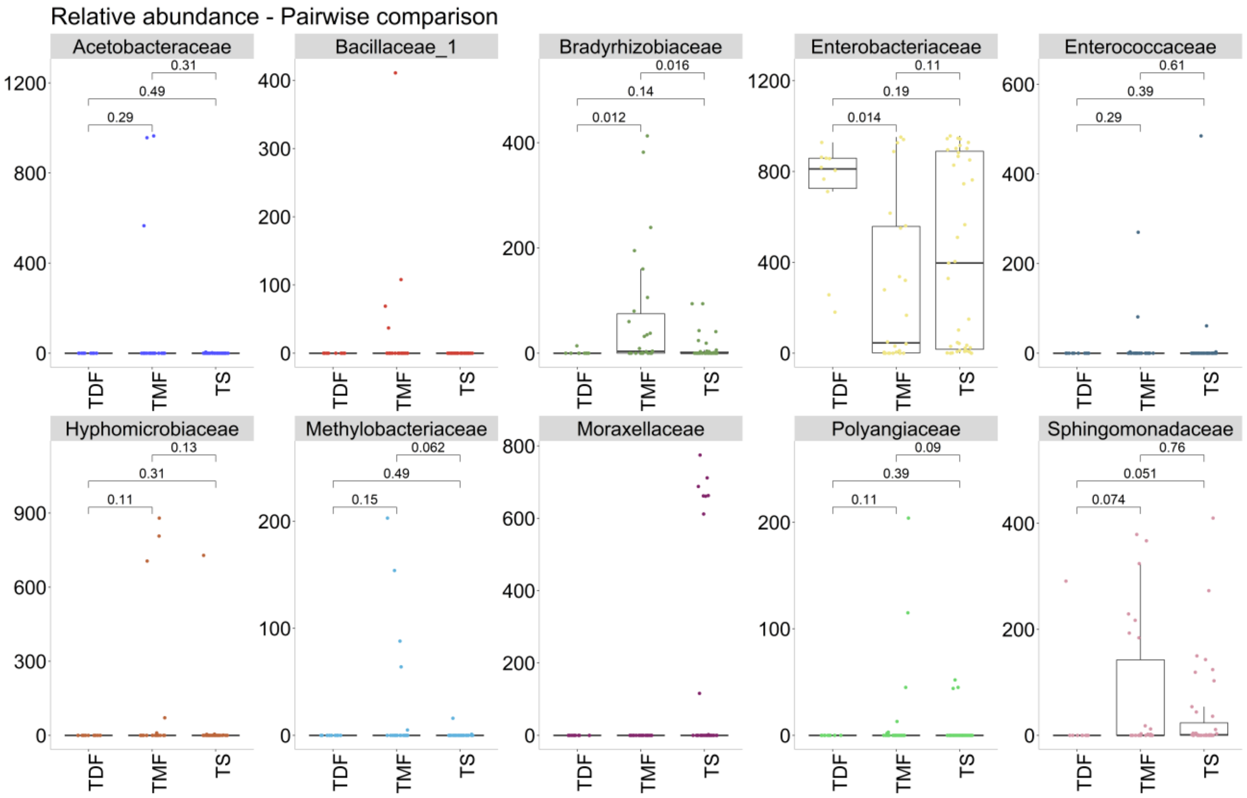

Figure 2. Abundance analysis of each bacteria family in the intestinal microbiome of *E. heros* collected in Brazil, classified by ecoregion. Pairwise comparison (Wilcoxon) is indicated between ecoregions.

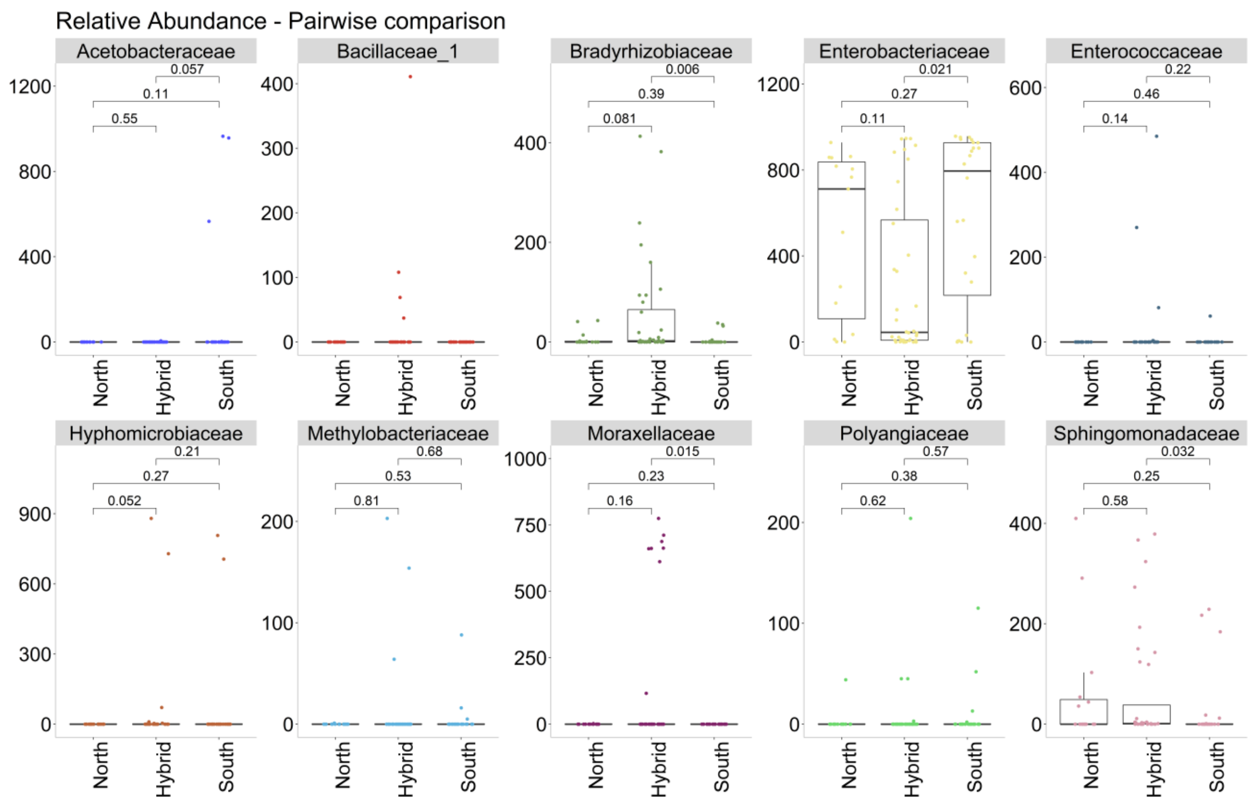

Figure 3. Abundance analysis of each bacteria family in the intestinal microbiome of *E. heros* collected in Brazil, classified by lineages. Pairwise comparison (Wilcoxon) is indicated between lineages.

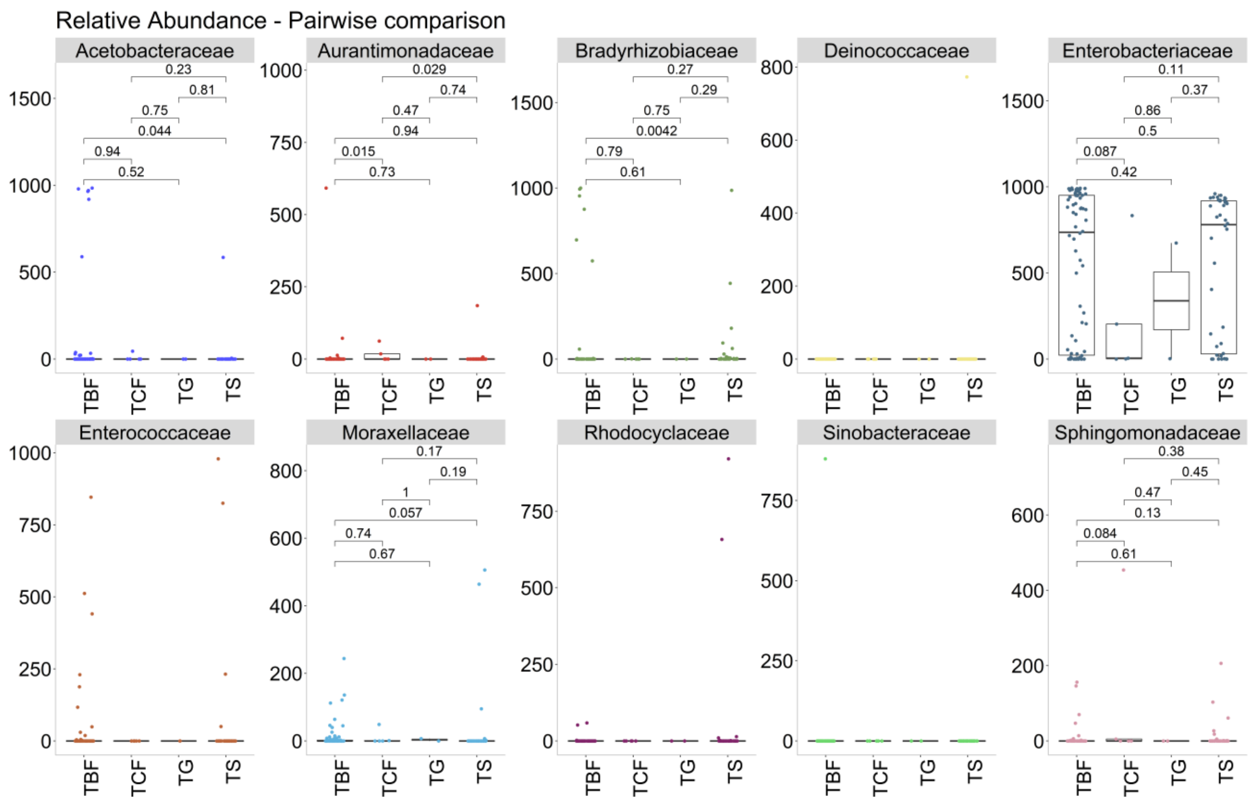

Figure 4. Abundance analysis of each bacteria family in the intestinal microbiome of *P. guildinii* collected in Brazil and the United States, classified by ecoregion. Pairwise comparison (Wilcoxon) is indicated between ecoregions.

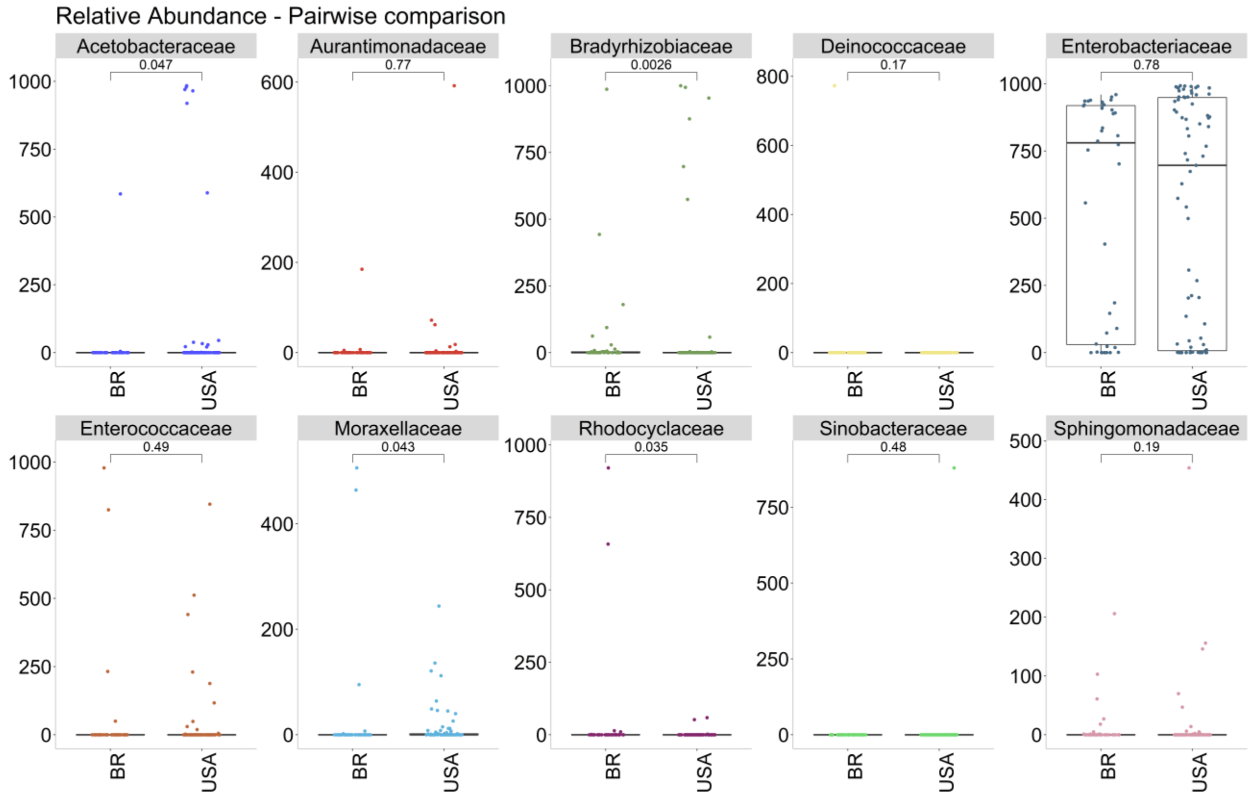

Figure 5. Abundance analysis of each bacteria family in the intestinal microbiome of *P. guildinii* collected in Brazil and the United States, classified by country. Pairwise comparison (Wilcoxon) is indicated between country.

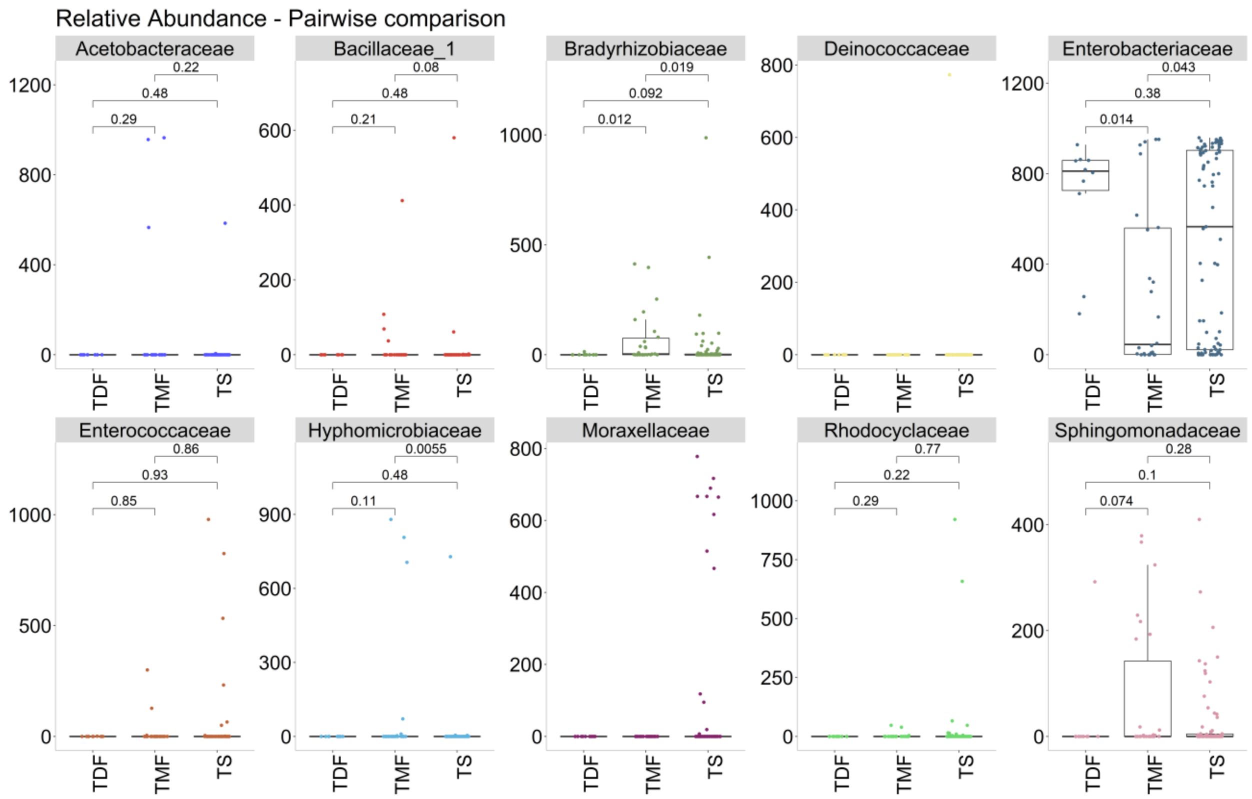

Figure 6. Abundance analysis of each bacteria family in the intestinal microbiome of *E. heros* and *P. guildinii* collected in Brazil, classified by ecoregions. Pairwise comparison (Wilcoxon) is indicated between ecoregions.

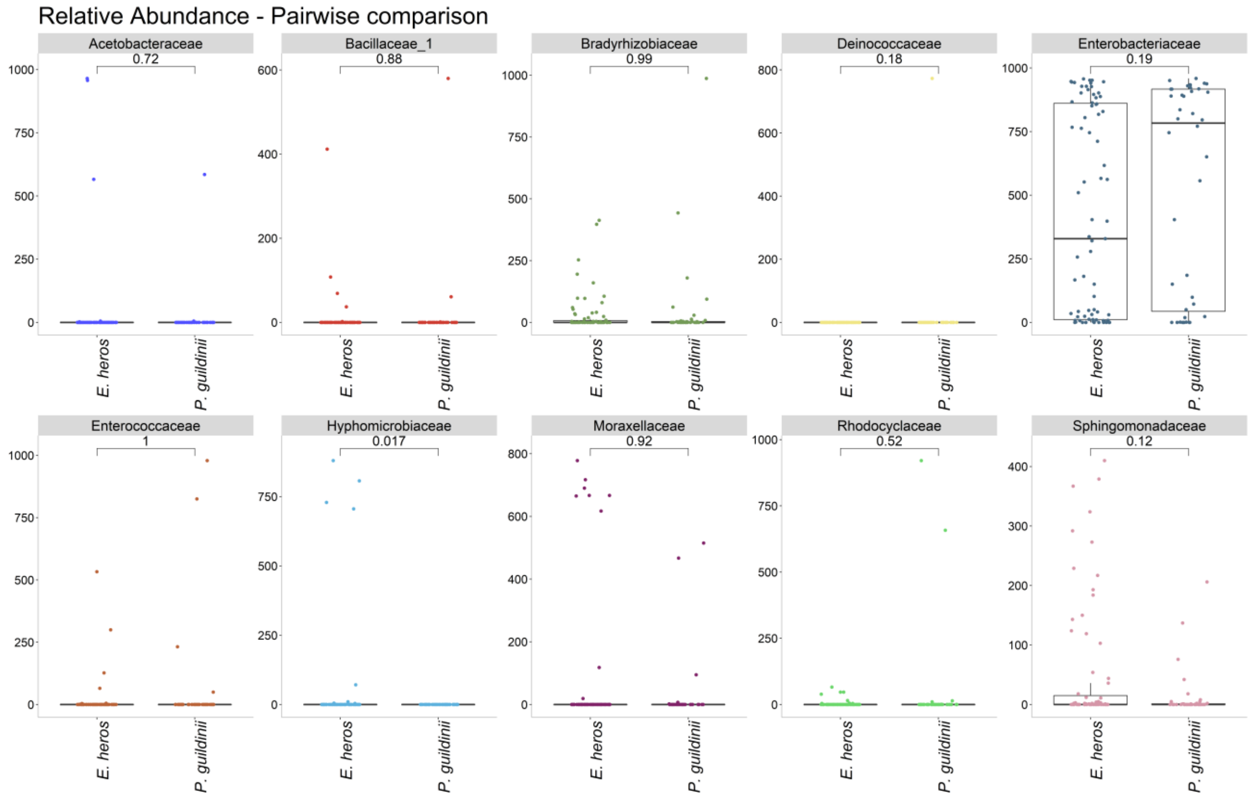

Figure 7. Abundance analysis of each bacteria family in the intestinal microbiome of *E. heros* and *P. guildinii* collected in Brazil, classified by species. Pairwise comparison (Wilcoxon) is indicated between species.
